# Primary non-Hodgkin's lymphoma of the infratemporal fossa: a rare case report

**DOI:** 10.1186/1758-3284-1-20

**Published:** 2009-06-21

**Authors:** Jagdeep S Thakur, Ravinder S Minhas, Narinder K Mohindroo, Dev R Sharma, Shobha Mohindroo, Anamika Thakur

**Affiliations:** 1 Department of Otolaryngology – Head & Neck Surgery, I. G. Medical College, Shimla, HP, 171001, India; 2 Department of Pathology, I. G. Medical College, Shimla, HP, 171001, India; 3 Department of Anatomy, I. G. Medical College, Shimla, HP, 171001, India; 4 Previous address: Dept of Pharmacology, I. G. Medical College, Shimla, HP, 171001, India

## Abstract

**Background:**

The head and neck are two of the most common sites of extranodal non-Hodgkin's lymphoma (NHL). However, primary tumors of the infratemporal fossa are infrequent, and NHL in this region is extremely rare.

**Case presentation:**

We present a case of a 41-year-old female that presented with swelling in the right preauricular region that had persisted for the past two years. The patient was diagnosed as having a small lymphocytic NHL. She initially underwent chemo-radiation but reported relapse. The tumor was excised and again the patient underwent chemotherapy. The patient remained symptomatic and developed a second primary squamous cell carcinoma in the right retromolar trigone.

**Discussion and conclusion:**

We discussed NHL with an emphasis on extranodal manifestations. Extranodal NHL that is limited to a single site can be managed by surgery and regular follow up. To the best of our knowledge, this is only the second case of primary NHL of the infratemporal fossa to be reported in the literature.

## Background

The head and neck are some of the most common sites of extranodal non-Hodgkin's lymphoma [1], with Waldeyer's ring being the most common site of manifestation within the region. The oral cavity, salivary glands, thyroid gland, paranasal sinuses, nasal cavity, parapharyngeal space, larynx, and infratemporal fossa are the other sites in which NHL may occur in the region [1-9]. However, primary tumors of the infratemporal fossa are infrequent, and NHL in this region is extremely rare [5-10]. In this report, we present a new case of extranodal NHL arising in the infratemporal fossa, the second such case to be reported in the literature. We reviewed the literature on non-Hodgkin lymphoma with a special emphasis on extranodal manifestations.

## Case presentation

In July 2006, a 41-year-old female presented with a swelling in the right preauricular region, which had persisted for the past two years, and was having difficulty opening her mouth for the past four months. The swelling was insidious in onset and progressive. In the first six months, the patient indicated the swelling was painless, only later becoming painful as the size increased.

**Figure 1:**
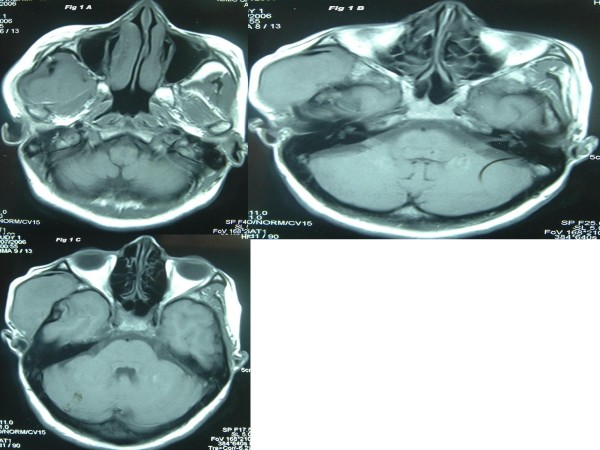
**A-C: MRI (axial sections) showing a big hypo-isointense mass in the right temporal and infratemporal fossa on T1 weighted images**.

**Figure 2:**
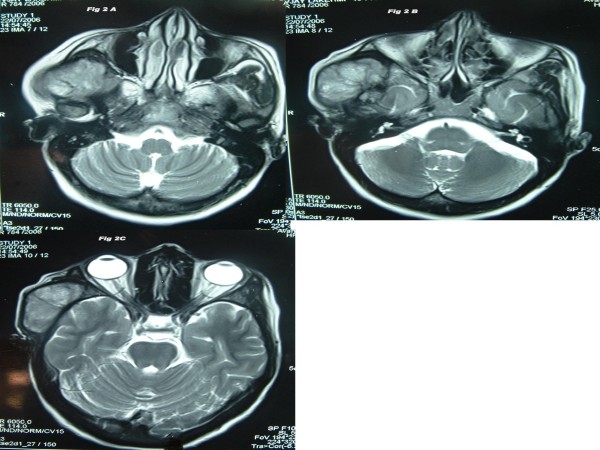
**A-C: This mass is heterogeneously hyper-intense on T2 weighted images (axial sections) of MR**.

**Figure 3:**
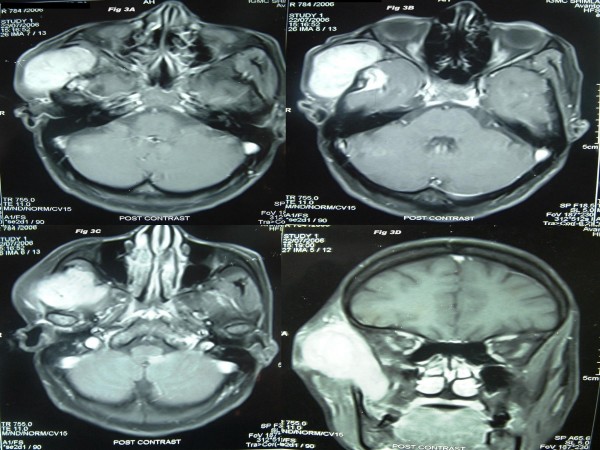
**A-D: The mass is showing significant enhancement in post-contrast MR images (axial and coronal sections)**.

**Figure 4:**
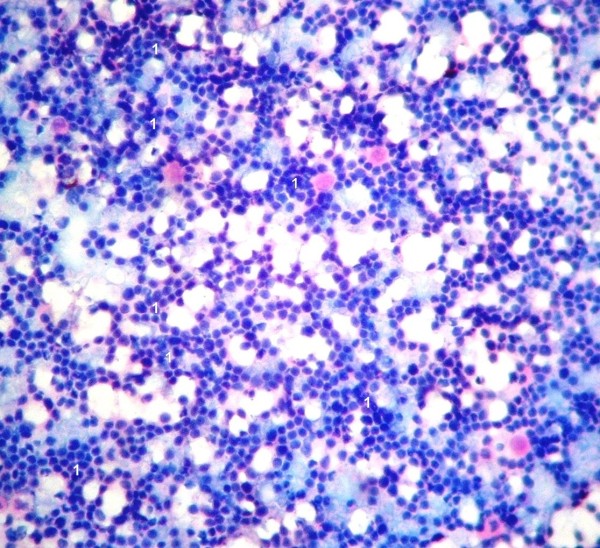
**Microphotograph of FNAC showing small, round lymphoid cells with regular nuclei, compact chromatin, inconspicuous nucleoli, and scant basophilic cytoplasm *(marked‘1’)* suggestive of NHL**. (MGG × 400).

**Figure 5:**
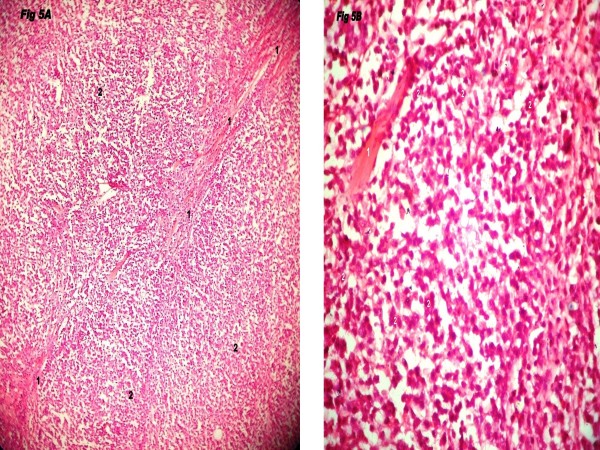
**A-B: Microphotograph of small lymphocytic NHL with infiltration into infratemporal fossa and showing hyalinised fibrous band (Marked '1') and small, round lymphoid cells with regular nuclei, compact chromatin, inconspicuous nucleoli, and scant basophilic cytoplasm (marked '2')**. (Fig 5A: H & E × 100; Fig 5B: H & E 400).

Local examination found a diffuse 5 × 4 cm firm to cystic mass with restricted mobility in the right preauricular region. Examination of the oral cavity, ear, cranial nerves, and other systems was unremarkable. MRI analysis indicated a large mass in the right infratemporal fossa with significant infiltration into the adjoining muscles. This mass was hypo-isointense on T1 (Fig 1A–C) and heterogeneously hyperintense on T2 weighted images (Fig 2A–C). The mass had significant enhancement in post-contrast MRI (Fig 3A–D). Hematological and biochemistry analyses were normal. Fine needle aspiration cytology (FNAC) (Fig. 4) revealed a monotonous population of small, round lymphoid cells with regular nuclei, compact chromatin, inconspicuous nucleoli, and scant basophilic cytoplasm. These findings were consistent with NHL. Diagnostic biopsy (Fig. 5A &5B) of the tissue confirmed small lymphocytic non-Hodgkin's lymphoma. The patient was investigated further to determine the staging of the NHL, but no lymph node or other organ was found to be involved. The patient was scheduled for chemo-radiation treatment and given nine cycles of the CHOP regime (cyclophosphamide, doxarubicine, vicristine, and prednisolone) and a total of 55G radiation in 25 fractions over five weeks. The patient remained asymptomatic for seven months.

**Figure 6:**
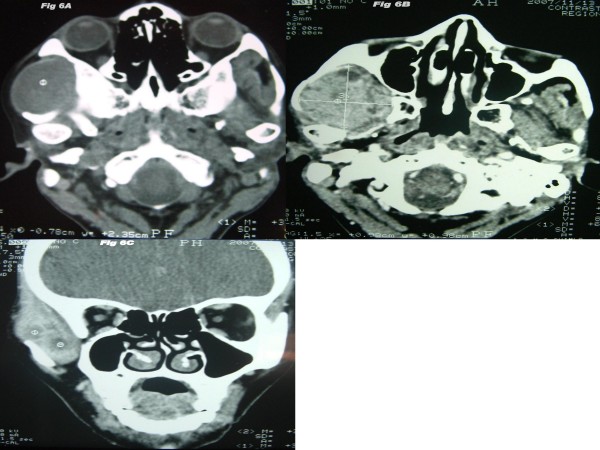
**A-C: CT scan (axial section) showing hypodense (37 HU) mass in the right temporal and infratemporal region (Fig. 6A)**. The mass had heterogeneous enhancement (66 HU) in contrast CT scan (axial and coronal sections) (Fig 6B-C). The adjoining muscles are indistinguishable from the mass.

**Figure 7:**
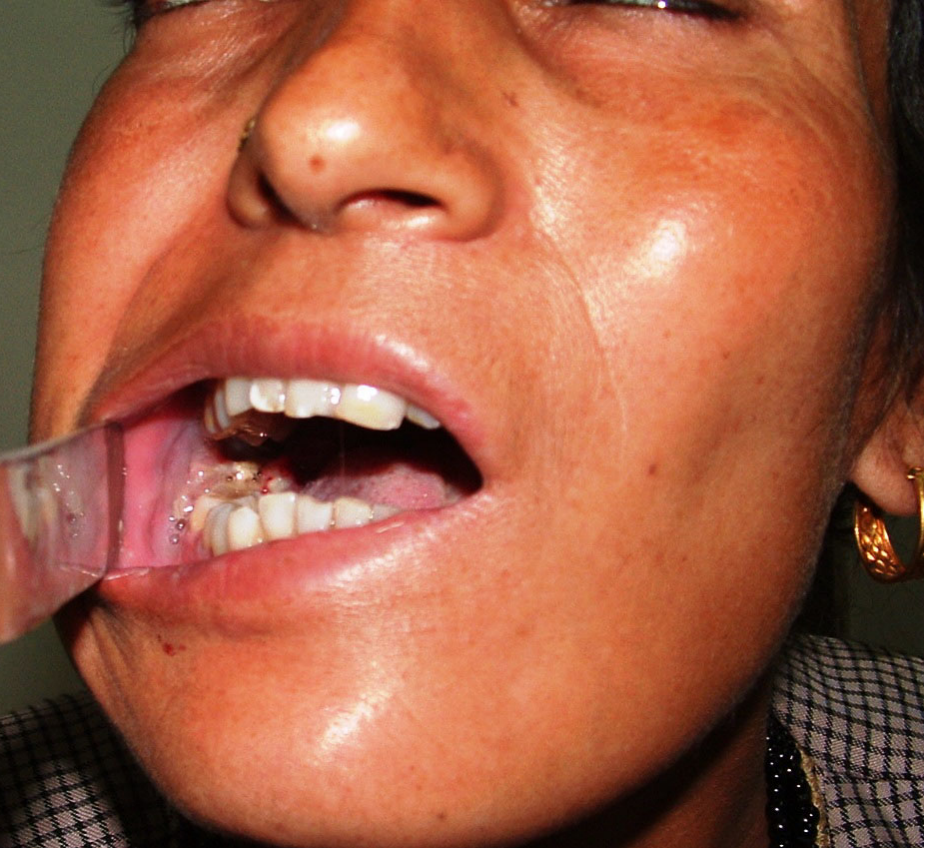
**Radiation induced second primary cancer in the oral cavity**.

In Nov 2007, the patient again presented with similar symptoms. A computed axial tomography (CT) scan (Fig. 6A–C) revealed a hypodense mass of 37 Hounsefield unit (HU) density and measuring 4.25 cm × 4.0 cm in the right temporal and infratemporal region. Post-contrast, this mass showed heterogeneous enhancement (66 HU density) and normal contents (muscles) were not identifiable from the mass. The tumor was excised and histopathology again confirmed the diagnosis of NHL. The patient was given six cycles of ifosfamide, metoxantron, and etoposide, with the last cycle on June 3^rd^, 2008. The patient was on regular follow up, and in Aug 2008 presented with increasing trismus. On examination, the infratemporal fossa was normal but there was a hard, irregular ulcer in the right retromolar area (Fig 7). A punch biopsy of the ulcer found it to be a well-differentiated squamous cell carcinoma. The patient was advised to undergo surgery for this carcinoma, but she did not come in for further follow up.

## Discussion

The gastrointestinal tract, followed by the head and neck, is the most common site of extranodal NHL [9], with the head and neck contributing to about 11% to 33% of total cases [11,12]. However, NHL is very rare in the superficial tissues of the head and neck region [8].

NHL is predominantly found in males of Caucasian descent [1,2,9,13]. Other risk factors [1,2,9,13-17] for the development of NHL include inherited and acquired immunodeficiency diseases, EBV, *H. pylori *and HTLV-I infections, chromosomal abnormalities, drug induced immunocompromised states, autoimmune diseases (e.g., rheumatoid arthritis and Sjogren's disease), exposure to pesticides and radiation, phenytoin therapy, previous history of Hodgkin's disease or chemotherapy, and excessive intake of meat and fat.

Extranodal NHL may present as a painless and progressively expanding lump or a widespread disease. The patient may be without any systemic symptoms ('A' symptoms) or may have night sweating, fever, and/or weight loss of greater than 10% of total body weight, which constitutes 'B' symptoms. FNAC gives a clear diagnosis of lymphoma and an uncrushed biopsy should be performed to ensure the accurate histological grading of the lymphoma [9].

In the head and neck, diffuse lymphoma (intermediate-grade) predominates, with diffuse large-cell lymphoma being the most common histological type of NHL [1,2,9]. B cells comprise the most common NHL of the head and neck, although T-cell lymphoma is common in the nasal cavity and nasopharynx [18,19].

The accurate staging of extranodal NHL is important for effective planning of the treatment approach [2]. Staging is determined by a detailed history, clinical examination, and radiological investigations. Conley *et al. *[2] found nodal disease in 50% of all cases at the time of presentation or during management, and they advised a detailed work up of all patients. It is further recommended that all cases of NHL should undergo a complete evaluation [1,8,9,13-17], including a complete hemogram, liver and renal functions, serum 2-microglobulions, chest X-ray, bone marrow biopsy, gallium scan, and cerebrospinal fluid analysis, as well as CT scans of the abdomen, pelvis, and bones. As a majority of our patients are low-income, initially we undertake ultrasonography of the abdomen and X-rays of the pelvis, vertebra, and femurs. Then, if required, a CT is performed in suspicious cases.

The Ann Arbor staging system classifies lymphoma in four stages (I-IV) with 'A' and 'B' symptoms and the recently added 'E' sub stage [15,17] for localized, solitary involvement of extra-lymphatic tissue, excluding the liver and bone marrow.

The treatment of NHL is a controversial issue [1,9,13-17,20]. It consists of chemotherapy, radiotherapy, or surgery. Sometimes, in cases of limited extranodal NHL, only surgery without chemotherapy or radiotherapy is used [17]. However, the main role of surgery is diagnostic only and rarely used for a cure [8], as in our case. In contrast, combined modalities, including surgery, chemotherapy, or radiotherapy, are used to treat advance NHL [13-17]. In stage I and II low grade extranodal lymphomas of the head and neck, radiotherapy is the first line of treatment, although the use of chemotherapy has also been advocated [1,9,13-17,20]. Chemotherapy is the standard treatment for intermediate grade lymphomas [13-17,20]. The addition of an anti-CD20 monoclonal antibody (rituximab) in the CHOP regimen (R-CHOP) has demonstrated promising results and is now frequently used for treatment of lymphomas. A total of 3–4 cycles of this regimen followed by radiotherapy are quite effective in the treatment of stage I and non-bulky stage II NHL; however, bulky stage II-IV tumors need 6–8 cycles of this chemotherapy. Bleomycin, etoposide, lomustine, carmustine, ifosfamide, and mitoxantrone are other chemotherapeutic agents used to treat NHL [17,20]. Advance stage and high grade NHL warrants aggressive chemotherapy and treatment is generally palliative. Autologous bone marrow transplantation is the treatment of choice for recurrent NHL that is still sensitive to chemotherapy. The use of radiolabeled, monoclonal antibodies (e.g., ibritumomab tiuxetan and tositumomab) and interferon have also been advocated, but clinical use is still limited [13-17,21].

Prognosis is calculated according to the International Prognostic Index (IPI) for patients at or above the age of 60 years. It is calculated based on age, LDH levels, performance status, and the number of extranodal sites involved. Factors such as molecular features of the tumor, levels of cytokines and soluble receptors, and surrogate markers have also been implicated in accurate prognosis, but these are clinically still under investigation [15]. The prognosis declines with an increase in the patient's age, LDH levels, histological grade, or stage of the NHL. According to the IPI, the 5-year survival for all ages is about 73%–83% for low grade, 51%–69% for low-intermediate, 43%–46% for high-intermediate, and 26%–32% for high-grade NHL [17]. Shima *et al. *[1] found decreased (28%) 5-years survival rates in young patients (≤ 20-years-old) irrespective of the stage of extranodal NHL.

A primary NHL of the infratemporal region is a rare clinical entity, and we found only one previous report of isolated NHL of the temporal region [8]. This report documented a 65-years-old female with painless temporal swelling. A complete excision was performed and the patient had no recurrence, even in the absence of chemo- or radiotherapy.

## Conclusion

This is the second case of primary NHL of the infratemporal fossa to be reported in the literature. The case discussed in this report was of clinical importance, as the patient had localized isolated primary NHL of the temporal region that was excised after failed chemoradiation. Histological examination found small lymphocytic-type NHL, which is infrequent in the head and neck region [1,2]. The patient had a complete recovery but subsequently presented with radiation-induced second primary squamous cell carcinoma. Limited extranodal NHL can be managed by surgery alone, and radiotherapy has only a limited efficacy and potential side effects, including the induction of a second primary tumor.

## Consent

Written informed consent was obtained from the patient for publication of this case report and any accompanying images. A copy of the written consent is available for review by the Editor-in-Chief of this journal.

## Competing interests

The authors declare that they have no competing interests.

## Authors' contributions

JST was the principal investigator and responsible for design, concept, drafting and writing of the paper. He takes the responsibility for the integrity of the article. RSM was involved with management of the case, concept, revision and final approval of the paper. NKM was involved with revision and final approval of the paper. DRS was involved with management of the patient, revision and final approval of the paper. SM was responsible for the histopathological analysis, critical revision and final approval of the paper. AT was involved in design, literature review, data collection, drafting and final approval of the paper. All authors read and approved the final manuscript.

JST is the principal author and takes responsibility for integrity of the article.
